# Getting Stronger Without Moving an Inch: A Randomized Controlled Trial Utilizing Maximal Isometric Co-Contraction

**DOI:** 10.3390/jfmk11020221

**Published:** 2026-05-29

**Authors:** Danny Lum, Paul Comfort, Dustin J. Oranchuk

**Affiliations:** 1 High Performance Sport Institute, Singapore 397630, Singapore; 2 Sport, Performance and Nutrition Research Group, School of Allied Health, Human Services and Sport, La Trobe University, Melbourne, VIC 3083, Australia; 3 Centre for Human Movement and Rehabilitation Science, School of Health and Society, University of Salford, Salford M5 4WT, UK; p.comfort@salford.ac.uk; 4 Department of Human Physiology & Nutrition, College of Nursing and Health Sciences, University of Colorado-Colorado Springs, Colorado Spring, CO 80918, USA; dustinoranchuk@gmail.com; 5 Muscle Morphology, Mechanics, and Performance Laboratory, Department of Physical Medicine and Rehabilitation, University of Colorado-Anschutz Medical Campus, Aurora, CO 80045, USA

**Keywords:** flexing, functional, holding, pushing, static, strength

## Abstract

**Background**: Maximal isometric co-contraction (MICC) of upper-limb muscles enhances strength and size, but its effects on lower-limb function are unknown. The aim of this study was to examine whether MICC training targeting the lower limbs can improve muscular strength and functional performance in sedentary adults. **Methods**: Twenty sedentary individuals (10 men, 10 women; 48.5 ± 7.5 years; BMI = 24.8 ± 4.2 kg/m^2^) were randomly assigned to either an experimental (EXP) or control (CON) group. The EXP group performed MICC of the knee flexors and extensors three times per week for four weeks, completing three sets of 5–10 maximal effort 3 s contractions per session. Assessments conducted pre- and post-intervention included the isometric mid-thigh pull (IMTP), 3-m timed up and go (TUG), and 30 s chair stand (CS) tests. **Results**: Significant time (*p* < 0.001) and time × group (*p* < 0.001) effects were found for all outcomes. Compared with CON, the EXP group showed greater improvements in IMTP peak force (*p* < 0.001, *g* = 1.80), faster TUG times (*p* < 0.001, *g* = 2.73), and more CS repetitions (*p* < 0.001, *g* = 3.90). **Conclusions**: Twelve sessions of MICC training improved maximal strength and functional performance in sedentary adults. This simple, equipment-free method may be particularly useful for individuals with limited access to conventional exercise facilities or supervision.

## 1. Introduction

Physiological changes, such as loss of muscle mass or quality, can impair physical function as people age [1,2,3]. One of the strongest predictors of adverse health outcomes associated with aging is poor lower-limb performance [4,5]. In addition, a study that included 1056 Singaporeans aged 40 and above reported that 9.6% of participants experienced falls [6]. Considering the importance of lower-limb strength in preventing falls [7], providing the general population with an effective and convenient lower-limb strength training method may increase participation rates and help reduce falls in the community. In addition, baseline exercise self-efficacy was reported to be an important determinant of exercise compliance [8], further reinforcing the need for convenient, simple training methods for the general population to perform.

Multiple studies have reported the benefits of strength training for improving physical function in older adults [9,10,11]. For example, Sipila et al. [11] reported that walking speed increased after nine and eighteen weeks of strength training with stack weights in older women. In addition, Katsura et al. [9] reported that 8 weeks of eccentric manual resistance training improved lower-limb strength, mobility, and postural stability in older adults. Although these findings show that strength training can improve function in older adults, the exercises used in these studies may require close supervision, limiting participation [12]. Hence, a simpler strength-training method would be beneficial for individuals who may not have regular contact with professionals to supervise them.

Isometric strength training is a mode of strength training that involves generating force without external movement [7,13,14]. This mode of strength training has been reported to benefit strength and dynamic movement performances [7,14]. In addition, isometric strength training can be performed without equipment [15,16,17]. Maeo et al. [15] reported that five sets of 10 repetitions with 4 s of maximal isometric co-contraction (MICC) per repetition of the elbow flexors and extensors training resulted in increased isometric strength after 4 and 12 weeks. These results were supported by Zbidi et al. [17], who reported that six sets of 8 repetitions with 5 s maximal MICC per repetition of elbow flexors and extensors training resulted in increased isometric strength and electromyographic activities after four and six weeks. However, while the authors also observed a 9.2–12.5% improvement in the rate of force development, it was not considered statistically significant. Despite showing that MICC of muscles can lead to increased muscular strength, no research groups have included any functional movement performance measures related to daily living [15]. Furthermore, no previous MICC training studies have utilized the training method on the lower body. Hence, it is unknown whether strength gains from MICC training would improve lower- and full-body strength and daily functional capacity.

Although Maeo and Kaneshisa [18] indicated that the MICC training method may not be effective for improving lower-limb muscular strength due to low muscle activity, no researchers have investigated its effects on lower-limb muscle function. Therefore, this study aimed to examine whether a short-term MICC training program, targeting the lower-limb muscles, could improve muscle strength and functional movement performance. We hypothesized that lower-limb MICC training would enhance functional performance related to daily living activities in sedentary adults.

## 2. Materials and Methods

### 2.1. Participants

Sample power was computed (G*Power, v.3.1.9.2, University of Kiel, Düsseldorf, Germany) assuming an expected large effect size (for instance, f = 0.4) [15], 5% of error probability for 95% of power, two groups, two measurements (i.e., pre- and post-test). Computation showed that a total sample size of *n* = 16 (i.e., *n* = 8 per group) was required to obtain a statistical power of 0.8.

We recruited 20 healthy sedentary adults for participation in the study. Of these, 10 were female (age: 47.5 ± 8.1 years, body-weight: 67.7 ± 9.7 kg, height: 161.1 ± 4.6), and 10 were male (age: 49.4 ± 7.2 years, body-weight: 70.4 ± 11.9 kg, height: 173.5 ± 4.5). This sample size was selected based on a priori sample size estimation and because previous researchers have found significant differences between intervention and control groups with smaller samples [15,17]. The inclusion criteria for the study are as follows: (1) male and female between 18 and 65 years of age; (2) have a sedentary lifestyle as characterized by less than 150 min of moderate intensity physical activity per week; and (3) not have any musculoskeletal injury, metabolic disease, cardiorespiratory diseases or any other chronic illnesses that would compromise their ability to participate in the study.

Before participation, all participants were briefed on the requirements and risks involved with the study. Participants were required to sign a written informed consent before the initial testing session. The study commenced after obtaining clearance from the Singapore Institute of Technology Institutional Review Board (Project number: 2023057, approved on 11 May 2023).

### 2.2. Testing Procedure

**Figure 1:**
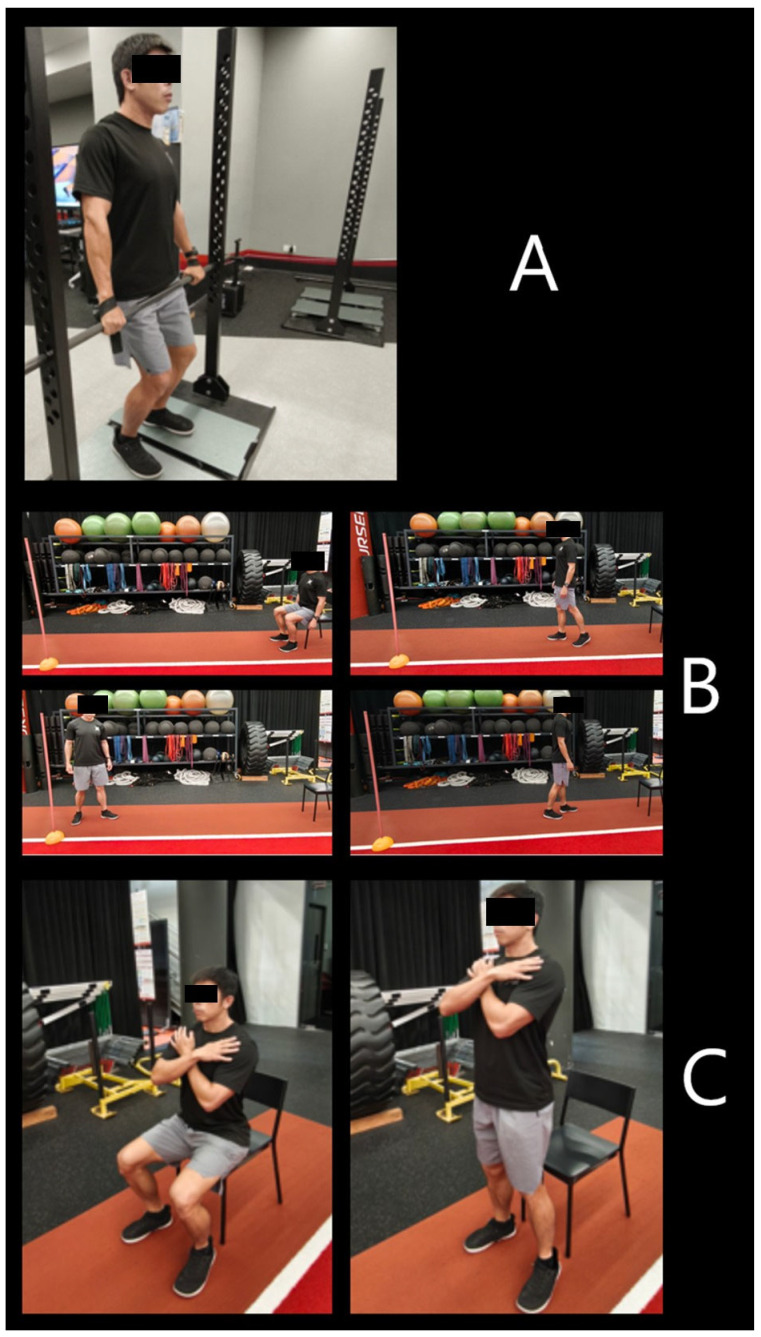
Pre- and post-intervention testing battery. (**A**) Isometric mid-thigh pull; (**B**) 3 m timed up-and-go; (**C**) 30 s chair stand. Isometric mid-thigh pull.

A randomized control trial research design was selected. Participants were recruited to complete one preliminary test session that included an isometric mid-thigh pull (IMTP), a 3-m timed up and go (TUG), and a 30 s chair stand (CS) (Figure 1). Subsequently, participants were matched for sex and then randomly assigned to either the control (CON) or the experimental (EXP) group. The EXP group completed 4 weeks of intervention training, 3 times per week. At the same time, CON continued their usual daily activities and were told to refrain from performing any lower-limb resistance exercise. Post-testing occurred between 72 and 96 h after the final training session and was conducted at the same time of the day as the preliminary testing session to account for diurnal effects.

Before all testing sessions, participants were required to refrain from consuming alcohol and caffeine for 24 h. Since coffee consumption was not measured in terms of the dosages of relevant compounds (e.g., mg of caffeine), the participants were told to continue with their usual habits of coffee drinking. All testing was conducted at the institution’s biomechanics lab. All training sessions were performed at the participants’ homes. All testing sessions commenced with a 5 min moderate-intensity (ratings of perceived exertion = 5) cycling on a cycling ergometer, followed by 10 repetitions of body-weight quarter squat, hip hinge, shoulder circumduction, and trunk rotation. A 1 min recovery period was provided before commencing the test.

Isometric strength testing is a valid and reliable method for assessing one of the key domains of muscle health, and is commonly used in clinical and sports performance settings [19]. Specifically, the IMTP is used to evaluate lower-body strength and is highly correlated with dynamic movements [20]. The IMTP was performed on the dual FP (Force Decks, VALD Performance, FD4000, Queensland, Australia), sampling at 1000 Hz. The commercially available ForceDecks software (VALD Performance, ForceDecks v2.2.0, Queensland, Australia) was used to analyze the force-time data, yielding an absolute gross peak force. A cold-steel bar used for the IMTP was mounted on a customized rig. Participants adopted a posture that reflects the start of the second pull of the weightlifting clean movement, resulting in a knee flexion angle of 125–145° and a hip flexion angle of 140–150° stance [21]. A handheld goniometer was used to ensure that participants adopted the required knee and hip angles. Participants were required to hold on to the bar with elbows fully extended. Hands were strapped to the bar to prevent grip strength from limiting performance. Before the test, participants performed a 3 s submaximal IMTP at 50%, 70% and 90% perceived maximal effort. Each repetition was separated by 60 s [21]. During the test, upon the tester’s command, “3, 2, 1 pull”, participants drove their feet into the floor, ‘as fast and as hard as possible’. Participants maintained the tension for 5 s. Participants performed each test twice, provided the peak force was within 5% between trials. Each attempt was separated by a 2 min recovery period [21]. The onset of pull was determined based on an increase of >5 standard deviations of the participant’s body mass during a period of quiet standing prior to the pull. The average of both trials was used for further analysis.

#### 2.2.1. 3-m Timed Up-and-Go, and 30 s Chair Stand

Both TUG and CS tests are commonly used in research and clinical settings [3] due to their high reliability and low cost. Both tests are valid means of assessing the functional performance domain of muscle health [19]. During the TUG, participants were required to stand from a chair, walk 3 m, walk back to the chair, and sit down [9]. The time taken to complete this process was measured with a stopwatch. Participants were required to perform the TUG twice, separated by a recovery period of 2 min. During the CS, participants sat in the middle of the chair with arms crossed at the chest. Participants were instructed to stand up and sit down as many times as possible within 30 s [9]. The number of repetitions completed in 30 s was recorded. Participants performed the CS twice, separated by a recovery period of 2 min. The average of both trials was used for further analysis. The average of both trials for each test was used for further analysis.

#### 2.2.2. Training

The CON group was instructed to continue their daily activities and refrain from any lower-limb strength training. The EXP group was required to continue their activities of daily living and to perform MICC of the lower limb three times per week for 4 weeks. In a seated position, with the buttocks positioned in the middle of the chair, knees at 90° and feet shoulder-width apart, participants performed maximal isometric co-contraction of the knee extensors and flexors. They were instructed to contract “as fast and as hard as possible” and sustain the contraction for a period of 3 s each repetition. The number of repetitions performed for each set was 5 (week 1), 7 (week 2), and 10 (weeks 3 and 4), with a total of 3 sets per session. The inter-repetition and inter-set rest times were 3 s and 2 min, respectively. All training sessions were performed at participants’ homes and supervised by the authors via video conference (Zoom, version 6.5.12). Participants completed all training sessions with no dropouts.

### 2.3. Data Analyses

All data were analyzed using JASP 0.19.3. All tested variables were expressed by Mean (±1 SD). Within-session test–retest reliability was assessed using two-way, mixed intraclass correlation coefficients (ICC) with the associated 95% confidence intervals (95%CI) and coefficient of variation (%CV) for all measured variables. ICC values were deemed as poor, moderate, good, or excellent if the lower bound 95%CI values were < 0.50, 0.50–0.74, 0.75–0.90, or >0.90, respectively [22]. Acceptable within-session variability was classified as <10% [23].

Repeated measure ANOVAs with Bonferroni post hoc analysis were performed for each selected variable. All assumptions to run ANOVA were checked beforehand, including normality and sphericity. A one-way ANOVA was used to determine the differences in performance change between groups. The associated 95%CI for the mean difference (MD) across all analyses was included. Whenever suitable and appropriate, Hedges’ g effect size was computed: (i) trivial effect size if g < 0.25; (ii) small effect size g = 0.25–0.50; (iii) moderate effect size if g = 0.51–1.00; (iv) large effect size if g > 1.00 [24].

## 3. Results

### 3.1. Reliability

There were no significant between-group differences for age (*p* = 0.841), body weight (*p* = 0.794), and height (*p* = 0.794). Test–retest reliability is summarized in Table 1; each test demonstrates excellent within-session reliability.

### 3.2. Isometric Mid-Thigh Pull

**Figure 2:**
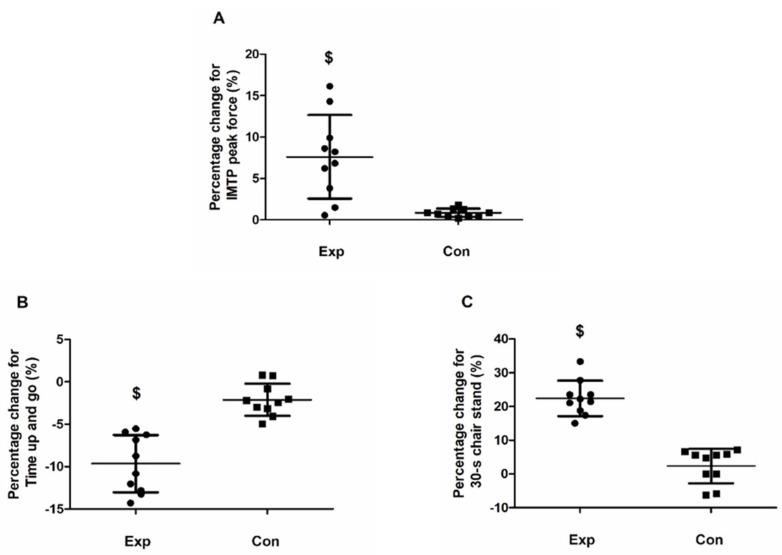
Group and individual percent changes for the co-contraction (EXP) and control (CON) interventions. (**A**) Isometric mid-thigh pull (IMTP); (**B**) Timed up-and-go; (**C**) 30-s chair stand. ^$^ Denotes statistically significant (*p* < 0.05) difference from CON.

There was no significant between-group difference for baseline IMTP peak force (*p* = 0.848, MD = 45.1 N, 95%CI: −441.4 to 531.6, *g* = 0.08). A significant time and time × group interaction effect was observed for IMTP peak force (Table 2). Significant yet small and trivial improvements in IMTP peak force were observed in EXP (*p* < 0.001, MD = 134.3 N, 95%CI: 77.6 to 191.1, *g* = 0.26) and CON (*p* < 0.001, MD = 15.2 N, 95%CI: 9.4 to 20.9, *g* = 0.03), respectively. EXP resulted in significantly and meaningfully greater improvement in IMTP peak force than CON (*p* < 0.001, MD = 6.7% 95%CI: 3.4 to 10.1, *g* = 1.78) (Figure 2A).

### 3.3. 3-m Timed Up-and-Go

There was no significant between-group difference for baseline TUG time (*p* = 0.860, MD = 0.04 s, 95%CI: −0.46 to 0.55, *g* = 0.07). A significant time and time × group interaction effect was observed for TUG time (Table 2). Significantly large and small improvements in TUG time were observed in EXP (*p* < 0.001, MD = −0.71 s, 95%CI: −0.92 to −0.50, *g* = 1.19) and CON (*p* = 0.007, MD = −0,15 s, 95%CI: −0.25 to −0.05, *g* = 0.39), respectively. EXP resulted in significantly and meaningfully greater improvement in TUG time than CON (*p* < 0.001, MD = −7.5% 95%CI: −10.1 to −5.0, *g* = 2.70) (Figure 2B).

### 3.4. 30 s Chair Stand

There was no significant between-group difference for baseline CS performance (*p* = 0.733, MD = 0.4 s, 95%CI: −2.0 to 2.8, *g* = 0.15). A significant time and time × group interaction effect was observed for CS performance (Table 2). A significant and large improvement in CS performance was observed in EXP (*p* < 0.001, MD = 3.9, 95%CI: 3.4 to 4.4, *g* = 1.41) but not in CON (*p* = 0.168, MD = 0.4, 95%CI: −0.2 to 1.0, *g* = 0.14). EXP resulted in significantly and meaningfully greater improvement in CS performance than CON (*p* < 0.001, MD = 20.1%, 95%CI: 15.2 to 24.9, *g* = 3.85) (Figure 2C).

## 4. Discussion

The present study was the first to examine the effects of lower-limb MICC training on physical function [16]. The main observation of the current study was that 12 sessions of MICC of the lower-limb muscles significantly increased muscle strength and functional movement performance. Hence, the hypothesis was supported by the current results, indicating MICC can be used as a training method to improve daily functions in sedentary individuals.

Previous researchers investigating the effect of MICC training on arm muscles reported improvements of 13–15% and 22.5–46% in arm flexor and extensor strength, respectively, and a 29.6–44% increase in electromyography activities after a 4–12-week intervention period [17,18]. In addition, Maeo & Kanehisa [18] also reported a significant increase in muscle thickness (4%) of both elbow flexors and extensors after 12 weeks but not after 4 weeks of intervention. Thus, indicating that strength improvement from MICC training in the short term (<12 weeks) was primarily attributed to neural adaptations. The magnitude of strength improvement in the current study (7.3%) was lower than that reported by Maeo & Kanehisa [18] and Zbidi et al. [17].

One possible reason could be the different methods used to assess muscular strength. The two earlier studies used a single joint isometric test, whereas the current study employed a multi-joint isometric test. The greater demand on muscle coordination and neural activity in the multi-joint isometric test, as compared to other tests, may have resulted in a lower overall improvement. Another possible reason could be that muscle activity during lower-limb MICC is relatively lower than during upper-limb MICC [18], although this was not evaluated in the present study. The lower magnitude of muscle activity during training may have reduced the stimulant required for greater strength adaptations. Nevertheless, the results showed that the MICC of the lower-limb muscles is efficacious in improving muscular strength within a relatively short period of time.

One limitation of previous studies on MICC training was the lack of assessment of functional movement [15,17]. The current study included the TUG and CS to assess whether MICC training of the lower limb could improve mobility in daily living. The results showed 9.6% and 22.4% in TUG and CS performance, respectively. The improvement in TUG and CS performance was likely because we were able to get up from the chair and walk faster due to increased muscular strength. This observation was consistent with the findings of Katsura et al. [9], who also reported concurrent improvements in lower-limb strength and in TUG and CS performance. Katsura et al. [9] reported improvements of 6.3–34.6% and 16.7–51% in TUG and CS performance, respectively, after 8 weeks of either a concentric or an eccentric contraction-focused home-based exercise intervention. While the relative improvement in the current results for the TUG was comparable to that observed by Katsura et al. [9] (0.8% per session vs. 0.8–2.1% per session), the relative improvement for CS was lower in the current study (1.9% per session vs. 4.3–6.4% per session). The likely reason for the lower magnitude of improvement in CS was that the intervention training by Katsura et al. [9] included the chair stand movement, thus allowing for specific neuromuscular adaptations to the movement assessed. Despite the lower magnitude of improvement, the current training method remained effective in enhancing functional movement performance. Furthermore, the current training method has the advantage of being simple and convenient for most people to perform.

Several limitations should be considered when interpreting the results of this study. Firstly, the participants in the study were mainly middle-aged to older sedentary adults, and the MICC exercise may result in different levels of adaptation in younger and more active individuals. Similarly, the effects of MICC have not been investigated in elderly or frail populations, which might benefit most from a simple, fast, and zero-equipment training method. Secondly, the extent of strength improvement in individual muscle groups is unknown, as a multi-joint isometric test was used to assess muscular strength. Furthermore, the intervention period lasted only 4 weeks. While it is promising to see substantial improvements in a brief window, it remains unknown how many additional adaptations will occur beyond this period. Finally, while muscle and physical performance were assessed, we did not include any measure of muscle size or body composition, the remaining muscle health domain [19].

## 5. Conclusions

Twelve sessions of MICC training for the lower limb increased muscular strength and performance of movement-related daily living tasks in sedentary adults. These findings provide individuals with convenient, straightforward strength-training options that may improve their lower-limb function. The results of this study may have implications for individuals sustaining musculoskeletal injuries and those requiring prolonged bed rest.

## Figures and Tables

**Table 1:** Within-session reliability data for all test measures.

Variables	ICC (95%CI)	%CV (95%CI)
IMTP peak force (N)	0.99 (0.94–1.00)	4.5 (2.9–9.3)
Timed up-and-go (s)	0.99 (0.97–1.00)	1.8 (1.2–3.7)
30 s chair stand (repetitions)	0.97 (0.92–0.99)	3.7 (2.7–5.7)

95%CI = 95% confidence interval, %CV = coefficient of variation, ICC = intraclass correlation coefficient, IMTP = isometric mid-thigh pull.

**Table 2:** Data of all performance measures.

Variables	EXP (*n* = 10)			CON (*n* = 10)			Time	Group	Time × Group
	Pre	Post	*g*	Pre	Post	*g*			
IMTP peak force (N)	1953.6 ± 510.0	2087.9 ± 495.9 *	0.26	1908.5 ± 525.4	1923.7 ± 524.7 ^#^	0.03	*p* < 0.001	*p* = 0.654	*p* < 0.001
Timed up-and-go (s)	7.22 ± 0.66	6.51 ± 0.47 *	1.19	7.18 ± 0.37	7.03 ± 0.37 ^#^	0.39	*p* < 0.001	*p* = 0.275	*p* < 0.001
30 s chair stand (repetitions)	17.7 ± 2.6	21.6 ± 2.7 *	1.41	17.3 ± 2.6	17.7 ± 2.8	0.14	*p* < 0.001	*p* = 0.084	*p* < 0.001

Expressed as mean (standard deviation) CON = control group, EXP = experimental group, IMTP = isometric mid-thigh pull. * Denotes significant difference from EXP Pre (*p* < 0.01). ^#^ Denotes significant difference from CON Pre (*p* < 0.01).

## Data Availability

The raw data supporting the conclusions of this article will be made available by the authors on request.

## Abbreviations

The following abbreviations are used in this manuscript:

BMI	Body Mass Index
CON	Control Group
CS	30 s Chair Stand
EXP	Experimental Group
IMTP	Isometric Mid-Thigh Pull
MICC	Maximal Isometric Co-Contraction
TUG	3 m Timed Up and Go
